# Microcarrier-Based Expansion of Adult Murine Side Population Stem Cells

**DOI:** 10.1371/journal.pone.0055187

**Published:** 2013-01-28

**Authors:** Christina A. Pacak, Mau-Thek Eddy, Lindsey Woodhull, Kai-Roy Wang, Ivan Alpatov, Shelby Fullen, Rory P. Dowd, Yeong-Hoon Choi, Douglas B. Cowan

**Affiliations:** 1 Department of Anesthesiology, Perioperative and Pain Medicine, Boston Children’s Hospital, Boston, Massachusetts, United States of America; 2 Department of Anæsthesia, Harvard Medical School, Boston, Massachusetts, United States of America; University of Houston, United States of America

## Abstract

The lack of reliable methods to efficiently isolate and propagate stem cell populations is a significant obstacle to the advancement of cell-based therapies for human diseases. One isolation technique is based on efflux of the fluorophore Hoechst 33342. Using fluorescence-activated cell sorting (FACS), a sub-population containing adult stem cells has been identified in a multitude of tissues in every mammalian species examined. These rare cells are referred to as the ‘side population’ or SP due to a distinctive FACS profile that results from weak staining by Hoechst dye. Although the SP contains multi-potent cells capable of differentiating toward hematopoietic and mesenchymal lineages; there is currently no method to efficiently expand them. Here, we describe a spinner-flask culture system containing C2C12 myoblasts attached to spherical microcarriers that act to support the growth of non-adherent, post-natal murine skeletal muscle and bone marrow SP cells. Using FACS and hemocytometry, we show expansion of unfractionated EGFP^+^ SP cells over 6 wks. A significant number of these cells retain characteristics of freshly-isolated, unfractionated SP cells with respect to protein expression and dye efflux capacity. Expansion of the SP will permit further study of these heterogeneous cells and determine their therapeutic potential for regenerative and reparative therapies.

## Introduction

The side population (SP) is a heterogeneous cell fraction that contains tissue-specific stem cells, which can be isolated from a multitude of tissues [1], [2], [3], [4], [5]. SP cells are also present as a sub-population in embryonic stem (ES) cell cultures, human solid tumors, and immortalized cell lines [6]. This fraction is identified by efflux of cell-permeant fluorescent dyes; an attribute that produces a characteristic profile upon analysis by cytometry [7], [8]. The ability to expel dyes from the cytoplasm results from expression of ATP-binding cassette proteins such as ABCG2 (also known as breast cancer resistance protein 1 [Bcrp1]) [9]. These proteins belong to a family of membrane-bound, multi-drug resistance transporters that discharge substrates into the extracellular space against steep concentration gradients in an energy dependent manner. The ability of SP cells to eliminate xenobiotic substances such as Hoechst 33342 and Rhodamine 123 as well as chemotherapeutic agents is believed to ensure their survival and likely accounts for their persistence in normal organs as well as in cancerous tissues and cell lines.

Since the discovery of SP cells, this population has been intensively investigated as a candidate for a multitude of regenerative and reparative therapies [5], [10], [11]. Despite this, it is apparent there are important problems to address before any therapy could become a clinical reality. In particular, the low rates of cell engraftment to target tissues, the inherent heterogeneity of the SP fraction, and a failure to expand these cells to a scale commensurate with a therapeutic application in humans continue to represent significant challenges. Establishing a reliable means to culture undifferentiated SP cells will permit comprehensive experimental evaluation and, ultimately, assessment of their therapeutic potential for a number of clinical applications.

To date, there have been few attempts to grow primary SP cells. In general, SP cells appear as non-adherent, small, circular cells with little proliferative capability. They are also predisposed to differentiate into multiple lineages upon attachment to a substrate [12]. If the SP fraction is derived from oncogenic cell lines or tumor cells, expansion in culture is feasible; though, the clinical utility of these cells is questionable as is their differentiation potential. Most experiments with cultured SP cells have been performed using viscous or semi-solid matrices to ascertain their hematopoietic potential [3]. These conditions promote differentiation and proliferation of hematopoietic cells allowing the progeny of single cells to form colonies of mature cells. While these cultures are optimized for outgrowth of hematopoietic colonies, they are of little utility for expansion of SP cells for regenerating or repairing solid tissues.

In this report, we describe a technique to grow unfractionated murine SP cells isolated from post-natal skeletal muscle and bone marrow. Non-adherent SP cells were expanded in suspension using paddle-impeller stirred flasks containing C2C12 myoblasts that were intended to function as support or ‘feeder’ cells, analogous to methods established to propagate ES cells [13]. At the same time, SP and C2C12 cells had different substrate attachment requirements; so, we seeded feeder cells on spherical microcarriers. With this approach, myoblasts were grown as monolayers on the surface of microcarriers and free-floating SP cells were added to suspension cultures [14]. The C2C12 line was chosen as a feeder cell to superficially resemble a muscle stem cell niche because we are primarily interested in developing cell-based therapies for skeletal muscle diseases. Here, we show expanded cells can be divided into two populations; one that closely resembles freshly-isolated SP cells and another that includes cells that are likely undergoing lineage commitment and differentiation.

## Materials and Methods

### Ethics Statement

Animal procedures were approved by the Institutional Animal Care and Use Committee at Boston Children’s Hospital (animal welfare assurance N^o^. A3303-01) and conducted according to the ‘Guide for the Care and Use of Laboratory Animals’ by the National Research Council.

### Isolation of SP Cells

Pooled skeletal muscle and whole bone marrow from femurs were collected and processed from 3 to 5 wk old male (♂) C57BL/6-Tg [ACTB-EGFP] 1Osb/J mice (Jackson Laboratories) [10]. For isolation of SP cells using flow cytometry, dissociated cells were filtered through a 40 µm cell strainer (Falcon), centrifuged (600 *g*) at 4°C for 10 min, and resuspended (10^6^ cells/mL) in phosphate buffered saline (PBS) containing 0.5% fraction V bovine serum albumin (BSA).

Hoechst 33342 (Sigma) was added at 5 µg/mL for bone marrow cells and 12.5 µg/mL for skeletal muscle cells [7], [8], [10]. In parallel, as a negative control for SP cell gating, 1×10^6^ cells were stained in the presence of 100 µM verapamil (Sigma). Staining took place for 90 min at 37°C and cells were washed with 5 volumes of 1×phosphate buffered saline-0.5% bovine serum albumin (PBS-BSA). Prior to cytometric analyses, cells were resuspended in PBS-0.5% BSA containing 2 µg/mL propidium iodide (PI) (Sigma) for 5 min. Cell sorting and analyses were performed using a FACSVantage SE system (BD Biosciences) using both a 488 nm argon ion laser at 200 mW (Coherent, Innova C) and a multi-line high UV 365 nm laser at 150 mW (Coherent, Innova 90-6) [7]. Hoechst and PI were both excited at 365 nm and emission signals were separated with a 500 short-pass dichroic mirror. Fluorescence emission was measured using 400 and 600 nm long-pass filters for Hoechst and PI, respectively. Enhanced green fluorescent protein (EGFP) was excited at 488 nm and emission signals passed through the same dichroic mirror and subsequently detected using a 530/30 band-pass filter. The sort head frequency was ∼26,000 Hz using the ‘normal sort’ mode and the sheath pressure was 11–12 psi. Using the conditions described by Montanaro *et al.* [7], cell viability, homogeneity, and yield was optimal for SP cells isolated from these tissues. A minimum of 20,000 live cell counts was acquired using CellQuest software (BD Biosciences) and then analyzed with FlowJo (Tree Star).

### SP Cell Expansion in Suspension Culture

Female (♀) C2C12 myoblasts (ATCC) were expanded on 150 mm plates (BD Biosciences) in high-glucose DMEM GlutaMAX with pyruvate (Life Technologies) containing 20% fetal bovine serum (FBS) (Atlanta Biologicals), 1% penicillin-streptomycin (Invitrogen), and 1% Fungizone (Invitrogen) prior to rinsing with PBS and detachment with 0.05% trypsin-EDTA (Life Technologies). Cells were collected by centrifugation at (600 *g*), resuspended in media and mixed with hydrated and sterilized Cytodex 1 microcarriers (Amersham Biosciences) on bacteriological Petri dishes (BD Biosciences). 1×10^6^ microcarriers were distributed to form a single layer and the use of Petri dishes promoted attachment to the Cytodex 1. Dishes were placed in a humidified culture incubator (Sanyo) with 5% CO_2_ and agitated every 15 min for 2 hr. C2C12 cells were seeded at a concentration of 50–100 cells per microcarrier. The cell-coated microcarriers were then divided into 2 Sigmacote (Sigma)-treated and sterilized 100 mL borosilicate glass spinner-flasks. Each flask contained two angled side-arms and an internal overhead-bearing impeller with a polytetrafluoroethylene paddle (Bellco Glass). Flasks were filled to 100 mL with media and the side-arm caps were loosened to allow for gas transfer. The flask contents were stirred at 30 rpm using a 5-position magnetic stirrer (Bellco Glass).

After 48–72 hr, the media was supplemented with 5 ng/L basic fibroblast growth factor (bFGF) (Life Technologies) and each flask was inoculated with freshly-isolated SP cells from skeletal muscle or bone marrow. Media in the flasks was replenished every 2–3 days by allowing the microcarriers and SP cells to settle for 10 min and then removing 50–70% of the media from the top of the flask and replacing it with freshly-prepared media containing 5 ng/L bFGF. Aliquots (5 mL) were collected from the side-arms at 3, 7, 10, 14, and 15 days for enumeration by hemocytometry and analysis by flow cytometry. Cultures were passaged 15 days after SP cell inoculation by collecting the contents of the flasks by gravity and dispersing the PBS-rinsed cell and microcarrier aggregates with 1% trypsin (Worthington) and agitation using a 37°C Enviro-genie incubator (Scientific Industries). Microcarriers were removed by passing the mixture through a 100 µM cell strainer (Falcon) and cells were collected by centrifugation and resuspended in media containing bFGF. These cells were added to prepared 250 mL spinner-flasks each containing 1×10^6^ microcarriers and cultured as described above. Cultures were maintained for 2 additional passages (*i.e.* P0 in a 100 mL flask, P1 in a 250 mL flask, and P2 in two 250 mL flasks, for each culture).

### Phenotyping and Growth Measurements

Throughout SP expansion, trypsinized aliquots were assessed for cell number and EGFP expression by a hemocytometer imaged with bright field and fluorescence illumination. Phenotypic characterization of cells was conducted by determining surface marker expression using flow cytometry [7]. Cultured cells were also assayed for intrinsic fluorescence and efflux of Hoechst using flow cytometry. Rat anti-mouse phycoerythrin (PE)-conjugated antibodies raised against CD45 (clone 30-F11), Sca-1 (clone E13-161.7), and IgG_2a,κ_ control antibodies (clone R35-95) (BD Biosciences) were used as described previously [7], [10]. Cells were washed with PBS-0.5% BSA, centrifuged, and resuspended in PBS-0.5% BSA and then analyzed as described earlier [7], [10]. For analysis of cells stained with fluorescently-conjugated primary antibodies using the FACSVantage SE system (BD Biosciences), EGFP and PE were excited with the 488-nm laser and emission signals were detected using either 530/30 or 575/25 band-pass filters [7]. Samples labeled with isotype-specific control antibodies were used to determine non-specific binding thresholds and to compensate for optimal separation of fluorescence.

### Staining of SP Cells

Aliquots from expansion cultures were fixed with 4% paraformaldehyde (PFA) in PBS for 1 hr at 4°C and, if detecting intracellular proteins, permeabilized for 3 min with 0.1% Triton X-100 in PBS. Microcarrier cultures or Shandon Cytospin (Thermo Scientific)-prepared slides of EGFP-positive (EGFP^+^) cells (3000 cells per slide) were used for immuno-staining. Primary antibodies were used at 1–10 µg/mL and incubated at 22°C for 30 min. In parallel, each preparation was incubated with a negative control antibody (Dako). The primary antibodies used include desmin (Sigma), myogenin (Dako), α-sarcomeric actin (Sigma), Pax7 (Developmental Studies Hybridoma Bank), MyoD (RDI), ABCG2 (Chemicon), α-actinin 2 [15] and Sca-1 (BD Biosciences). For detection of antibodies not fluorescently-conjugated, species-appropriate Alexa-conjugated secondary antibodies (Life Technologies) were used and mixed with 4′, 6-diamidino-2-phenylindole dihydrochloride (DAPI) (Life Technologies). In some cases, Texas Red-X phalloidin (Life Technologies) was used to stain the actin cytoskeleton (*i.e.* filamentous [F]-actin). Cells were visualized on a CARV spinning-disk confocal system (Atto Bioscience) attached to an Axiovert 200M microscope (Carl Zeiss). Images were acquired with a CoolSNAP HQ camera (Photometrics) controlled with MetaMorph 6.2 software (Universal Imaging). For scanning electron microscopy (SEM), microcarrier cultures were washed in PBS and fixed in 2.5% grade I glutaraldehyde suspended in 0.1 M cacodylate buffer. Sample preparation and SEM imaging was performed by Dennis Kunkel Microscopy, Inc. (Kailua, HI).

### Data Analysis

Statistical analyses were performed using InStat software (GraphPad). Data is presented as mean (standard deviation [SD]) and significance was determined by Student’s *t* test.

## Results and Discussion

In this report, we describe a stirred-flask suspension culture system adapted to expand unfractionated SP stem cells isolated from adult skeletal muscle and bone marrow. To expedite development of the expansion system, we used murine cells in order to isolate fluorescent SP cells from ♂ C57BL/6-Tg [ACTB-EGFP] 1Osb/J mice and co-culture these with unlabeled ♀ C2C12 cells. Our previous attempts to culture SP cells in suspension without the presence of supporting feeder cells resulted in no proliferation of this population; however, another group has reported the co-culture of murine skeletal muscle SP cells with primary myoblasts, C2C12 myoblasts, and C3H10T1/2 fibroblasts [2]. Although their goal was to examine the differentiation potential of SP cells rather than to expand this population, Asakura *et al.* demonstrated myogenic specification of SP cells in the presence of primary or C2C12 myoblasts, but not with the fibroblast cell line or when cultured alone using conditioned medium from primary myoblast cultures [2]. Because we are interested in developing cell-based therapies for skeletal muscle diseases, we used this information to help design our expansion system.

Rather than simply mixing cells together in a conventional culture plate, we reasoned a 3-dimensional bioreactor would produce better cell yields and allow for isolation of the SP from co-cultures for subsequent directed differentiation and transplantation studies. Consequently, adherent, non-fluorescent ♀ C2C12 cells were seeded onto Cytodex 1 microcarriers to function as a feeder cell layer. This culture strategy permited determination of the disposition of the ♂ EGFP^+^ SP cells by intrinsic fluorescence, antibody reactivity, or if necessary, Y chromosome staining using fluorescence *in situ* hybridization. We found skeletal muscle and bone marrow SP expansion through each of 3 passages and a proportion of the EGFP^+^ cells retained attributes of freshly-isolated SP stem cells with respect to surface markers, protein expression, and Hoechst dye efflux. The ability to generate large numbers of SP cells will allow for further experimental characterization and, ultimately, determine the regenerative and reparative potential of these adult tissue-specific stem cells.

**Figure 1:**
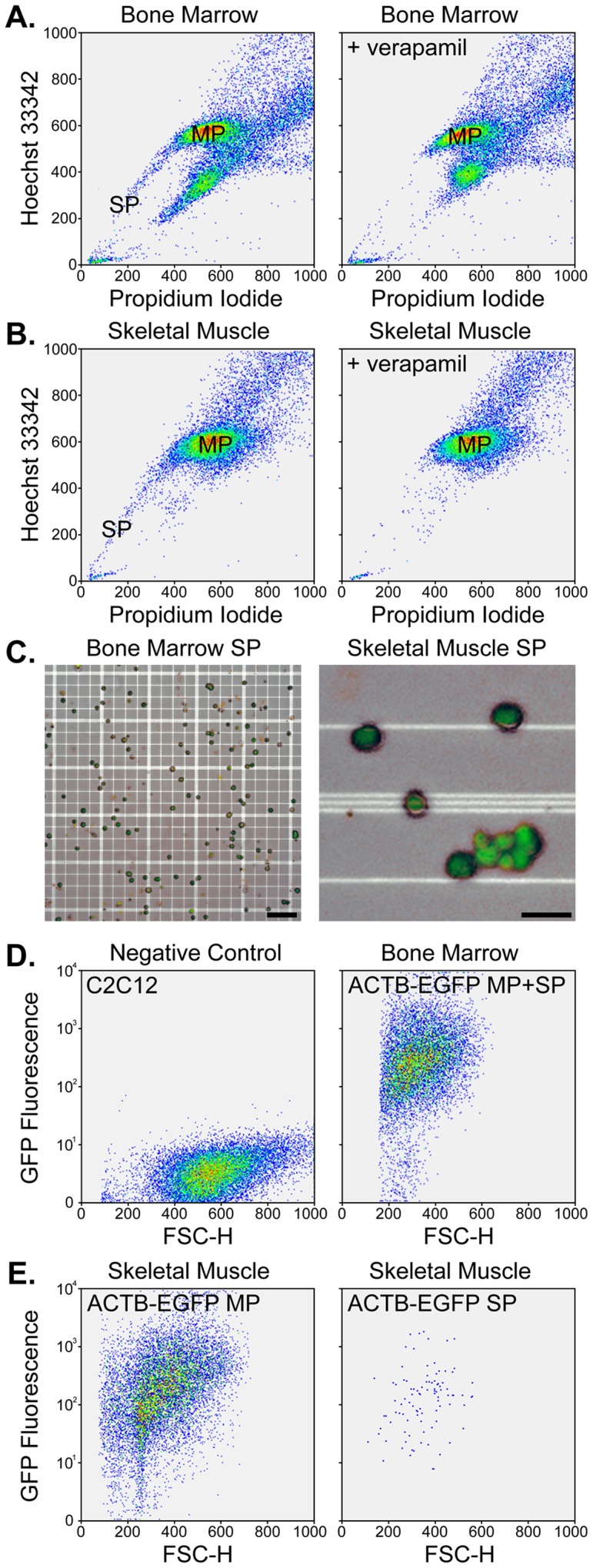
Isolation of SP cells from murine bone marrow and skeletal muscle. A – Representative FACS plots of bone marrow cells stained with Hoechst 33342 and PI. All plots show 20,000 events. Addition of verapamil eliminated the SP. The linear X and Y axes show PI and Hoechst staining, respectively. B – Representative FACS plots of skeletal muscle cells stained with Hoechst 33342 and PI (20,000 events per plot) in the absence (left) or presence (right) of verapamil. C – The appearance of bone marrow (left) and skeletal muscle (right) SP cells under combined bright field and fluorescence illumination. EGFP-expressing SP cells are shown on a hemocytometer grid and scale bars represent 50 µm (left) and 10 µm (right). D – Representative intrinsic fluorescence in C2C12 cells and unfractionated bone marrow from EGFP-expressing mice. The X axis depicts forward scatter (FSC-H) using a linear scale and the Y axis shows fluorescence using a logarithmic scale. E – Representative fluorescence in MP and SP fractions in enzymatically-digested skeletal muscle from ACTB-EGFP transgenic mice.

**Figure 2:**
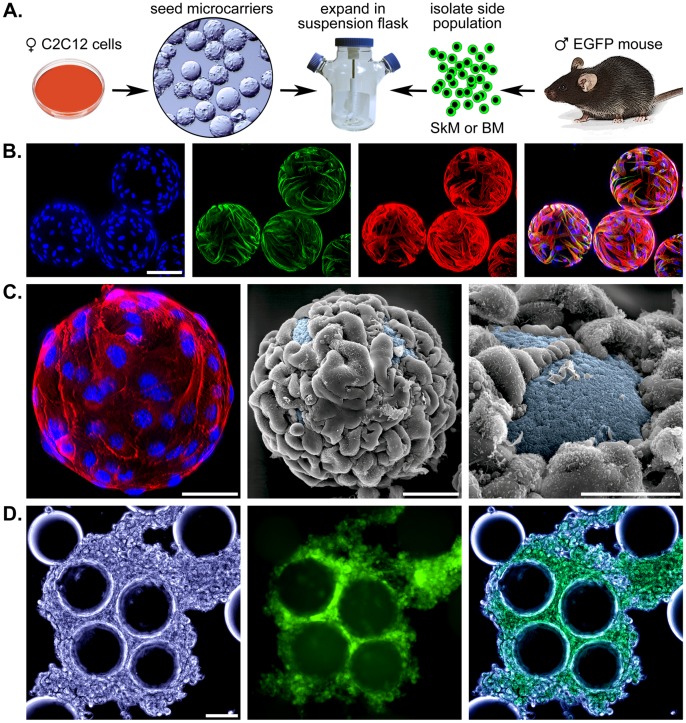
Appearance of microcarrier-based cultures from stirred-flasks. A – A schematic representation of the initial steps in establishing a SP expansion culture. ♀ C2C12 myoblasts are expanded and seeded on 500,000 Cytodex 1 microcarriers (left). After attachment to the microcarrier surface, cultures are transferred to 100 mL flasks and maintained for 2–3 days. Freshly-isolated bone marrow (BM) or skeletal muscle (SkM) SP cells isolated from ♂ EGFP-expressing mice are added and stirred at 30 rpm. B – Fluorescent staining of C2C12-containing Cytodex 1 microcarriers using DAPI (blue), an α-sarcomeric actin antibody detected with a conjugated secondary antibody (green), and Texas Red-X phalloidin (red). The combined image is on the right and the scale bar represents 100 µm. C – A closer view of a cellularized microcarrier stained for F-actin and DNA (left) as well as 2 scanning electron micrographs (SEMs). The SEMs were pseudo-colored to emphasize the dextran matrix on the microcarrier surface. Scale bars equal 100 µm (left and center) and 50 µm (right). D – Differential interference contrast, fluorescence, and combined images of day 18 co-cultures of SP cells from bone marrow and myoblast feeder cells. Scale bar equals 100 µm.

We began our studies by isolating SP cells from bone marrow (Fig. 1A) and skeletal muscle (Fig. 1B) [7], [8]. Using established Hoechst concentrations, we observed a typical SP profile when cytometry data was presented as a two-dimensional plot for each tissue. The SP fraction was reduced by addition of the calcium channel blocker verapamil due to inhibition of ATP-binding cassette transporter activity [7], [8]. Examination of SP cells revealed small fluorescent cells that appeared indistinguishable regardless of their tissue source (Fig. 1C). Because we are interested in developing therapies for muscle diseases, to standardize and simplify development of the expansion system, we chose to co-culture SP cells with non-fluorescent C2C12 myoblasts (Fig. 1D). Cells isolated from bone marrow or skeletal muscle of ACTB-EGFP mice were confirmed to be fluorescent regardless of whether they comprised the SP or ‘main population’ (MP) (Fig. 1E). These results demonstrate proficient isolation of EGFP^+^ SP cells from murine bone marrow or skeletal muscle and further shows the latter cells are easily distinguished from non-fluorescent C2C12 myoblasts. This distinction served as a starting point for development of an co-culture expansion system (Fig. 2).

The workflow for initiating this system is depicted in Fig. 2A. Briefly, C2C12 cells were expanded in standard culture plates and then seeded on Cytodex 1 microcarriers, which are composed of cross-linked dextran in a spherical matrix. After static seeding to allow cells to adhere to the microcarrier surface, cultures were maintained at 30 rpm in spinner-flasks. Each 100 mL flask contained 500,000 Cytodex 1 microcarriers and most spheres had about 100 cells on their surface (*i.e.* 5×10^7^ C2C12 cells/100 mL stirred-flask). By the time bFGF was added to the media to support SP cell expansion, the microcarriers were completely covered and the myoblasts had begun to spread and fuse with one another. Immuno-staining showed extensive coverage of the Cytodex surface with cells expressing α-sarcomeric actin and F-actin (Figs. 2B and 2C). Skeletal muscle and bone marrow EGFP^+^ SP cells were added to flasks and cultures were intermittently assessed for propagation. We found DMEM with 20% FBS plus 5 ng/mL bFGF brought about a dramatic rise in the number of EGFP^+^ cells after 2 wks (Fig. 2D).

One issue that became apparent during the course of these experiments was the loss of feeder cells from the microcarrier surface in cultures maintained for greater than 15 days (Fig. 2D). This phenomenon was likely due to prolonged exposure to shear stress and resulted in a decline in EGFP^+^ cell expansion. To avoid this problem, we passaged cells every 2 wks. In this way, single 100 mL stirrer flasks for bone marrow or skeletal muscle SP cell expansion (P0) were transferred to 250 mL flasks (P1) containing freshly-seeded microcarriers. P1 cultures were then used to inoculate a pair of 250 mL flasks (P2). While this process allowed for expansion of the EGFP^+^ fraction, we found that cell proliferation was not completely scalable as passaging to a 1000 mL stirrer flask caused a collapse in the EGFP^+^ cell population. This probably resulted from inadequate gas transfer and may be rectified by introducing gases directly into stirrer flasks rather than relying on passive diffusion. We were also anticipating that non-adherent SP cells could be effortlessly separated from feeder cells attached to the microcarriers by allowing the Cytodex spheres to sink to the bottom of the flasks or filtering them out with a cell strainer and collecting free-floating cells. We did not observe segregation of non-fluorescent cells from EGFP^+^ cells. Over time, fluorescent cells were attached to or clustered around the microcarriers; so, separation of expanded cells required a cell sorter.

**Figure 3:**
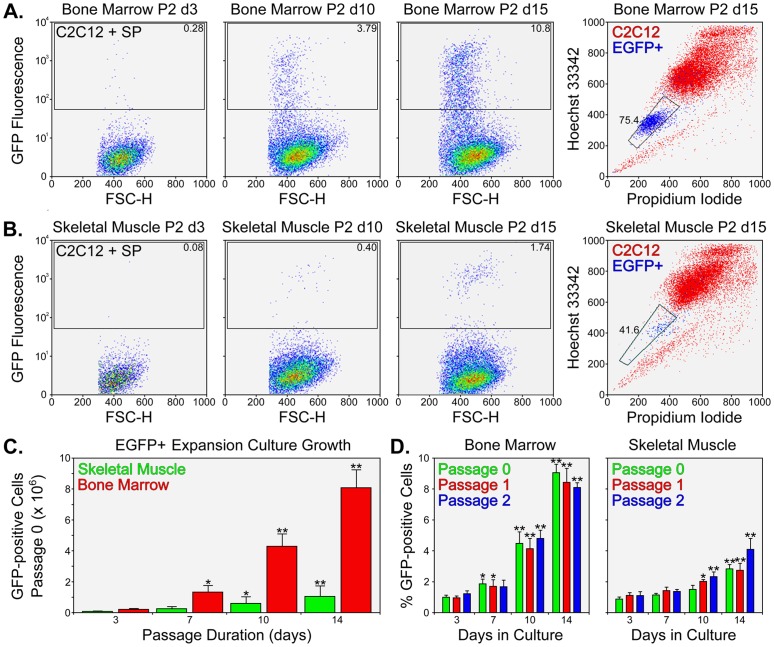
Expansion of bone marrow and skeletal muscle SP cells in suspension. A – Representative FACS plots of P2 bone marrow suspension culture analyses for EGFP-fluorescence at days 3, 10, and 15 (left). Plots show 20,000 events. Day 15 cells were assessed for Hoechst dye effusion (right). Plots show 50,000 events. B – FACS plots of P2 skeletal muscle culture analyses for EGFP-fluorescence at days 3, 10, and 15 (left). Day 15 cells were assessed for Hoechst dye effusion (right). Both bone marrow and skeletal muscle expansion cultures demonstrated an increase in EGFP^+^ cells over time and many cells were Hoechst^low^, analogous to freshly-isolated SP cells. C – Expansion of EGFP^+^ cells in P0 stirred-flask cultures as determined by hemocytometry. Numerical values for the total number of EGFP^+^ cells are expressed as Mean ± SD (*n* = 4) and asterisks represent statistical significance (* P<0.05 and ** P<0.01) compared to day 3 values. D – Expansion of bone marrow (left) and skeletal muscle (right) EGFP^+^ cells over the course of 3 passages (P0, P1, and P2). The percentage of EGFP^+^ cells in the suspension cultures is expressed as Mean ± SD (*n* = 4) and asterisks represent statistical significance (* P<0.05 and ** P<0.01) compared to day 3 values.

To define the growth of EGFP^+^ cells in suspension, we obtained aliquots of cells at different times following the initial inoculation of SP cells. Expanded cells were enumerated by hemocytometry and characterized for Hoechst dye efflux and EGFP fluorescence by FACS (Fig. 3). Cytometric analyses were performed in 4 independent cultures for 3 passages (P0, P1, and P2). Typical FACS plots for P2 bone marrow (Fig. 3A) and skeletal muscle (Fig. 3B) cultures show increases in EGFP^+^ cells over time. Intriguingly, many of these cells eliminated Hoechst dye comparable to freshly-isolated SP cells, whereas other cells sorted to the MP position. In other words, for a typical assay of skeletal muscle from a single mouse, we acquired 14,885.5 (7,885.7) (Mean [SD]) viable skeletal muscle SP cells and 41,874.3 (21,642.2) (Mean [SD]) bone marrow SP cells from a single EGFP-expressing mouse. The significant rise in EGFP^+^ cells in P0 cultures is depicted in Fig. 3C, which clearly shows better expansion of bone marrow cultures compared to skeletal muscle cultures. In bone marrow SP expansion cultures, the number of EGFP^+^ cells increased from approximately 4.2×10^4^ to 8.1×10^6^ (Fig. 3C) in the P0 cultures, which represents an estimated doubling time of 1.8 days. In comparison, skeletal muscle EGFP^+^ expansion cultures had a doubling time of 2.3 days. Similar growth rates were observed in P1 and P2 suspension cultures inoculated with comparable numbers of EGFP^+^ cells as P0 cultures (Fig. 3D). Together these results point toward a continuous and reproducible expansion of SP-derived EGFP^+^ cells over a period of 6 wks.

**Figure 4:**
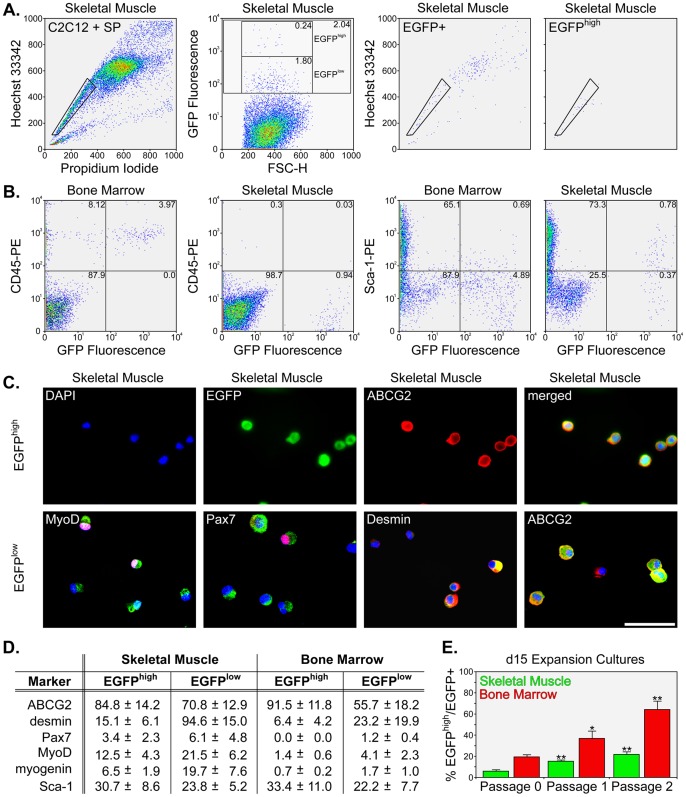
Phenotypic characterization of stirred-flask expansion cultures. A – Representative FACS plots of P0 skeletal muscle expansion cultures. The increase in the typical SP gate position in day 15 cultures is shown (left). The plot shows 50,000 events. By day 10, the appearance of a highly-fluorescent (EGFP^high^) sub-population was apparent in EGFP^+^ cells and these were found to sort nearly exclusively to the SP gate (right). The EGFP^low^ cells were distributed in typical SP and MP positions. B – Representative FACS analyses of surface marker expression in P2 bone marrow and P3 skeletal muscle cultures. Plots show 20,000 events. CD45 (left) and Sca-1 (right) profiles plotted against GFP fluorescence are shown for unfractionated aliquots of the suspension cultures. C – Representative immuno-staining results from Cytospin preparations for the EGFP^high^ (top) and EGFP^low^ (bottom) sub-populations from skeletal muscle. The EGFP^high^ images show separate channels depicting DNA staining, GFP fluorescence, ABCG2 immuno-staining, and a combination of fluorescent channels (left to right). EGFP^low^ images represent the same channels except the red channel shows immuno-staining with MyoD, Pax7, desmin, and ABCG2 (left to right). Scale bars equal 25 µm. D – A summary of immuno-staining experiments for ABCG2, desmin, Pax7, MyoD, myogenin, and Sca-1 in EGFP^high^ and EGFP^low^ sub-populations. Percentage values are expressed as Mean ± SD (*n* = 6). E – A graph showing the increase in the EGFP^high^ sub-population in skeletal muscle and bone marrow cultures at the end of P0, P1, and P2. The percentage of EGFP^high^ cells in the EGFP^+^ fraction is expressed as Mean ± SD (*n* = 5) and asterisks represent statistical significance (* P<0.05 and ** P<0.01) compared to day 15 P0 values.

Additional FACS analyses of the expansion cultures established the EGFP^+^ fraction exhibited variable green fluorescence emission that could be exploited to isolate highly-purified SP cells by excluding cells that underwent a decrease in Hoechst efflux (Fig. 4). We observed EGFP^+^ cells could be distinguished as intensely or weakly fluorescent in both bone marrow (not shown) and skeletal muscle cultures (Fig. 4A). Highly-fluorescent EGFP^+^ cells, which we termed the EGFP^high^ population, displayed a nearly identical Hoechst 33342 FACS profile as freshly-isolated SP cells. In contrast, weakly fluorescent EGFP^+^ cells (termed the EGFP^low^ fraction) showed a similar distribution of Hoechst/PI-stained cells as expanded cells did when determined *en masse*. As a result, we identified a means to distinguish expanded SP cells from mixed populations of EGFP^+^ SP and SP-derived cells.

Verification that the EGFP^high^ and EGFP^low^ fractions represented the SP and a mixed population of SP as well as ‘SP-derived’ MP cells, respectively, was achieved by antibody staining and analysis by flow cytometry and fluorescence microscopy. The tyrosine phosphatase CD45 is located on many hematopoietic cells and is often used as a marker for bone marrow, whereas Sca-1 (Ly-6A/E) is variably expressed by stem and progenitor cell populations. Cytometric analyses of these markers along with EGFP fluorescence is shown in Fig. 4B. Similar to what is observed in freshly-isolated SP cells, bone marrow EGFP^+^ cells were positive for CD45 while none of the skeletal muscle EGFP^+^ cells expressed this marker [1], [7], [16]. On the other hand, Sca-1 was positive in 22.4% (4.8) (Mean [SD], *n* = 3) of bone marrow EGFP^+^ cells and 30.1% (9.2) (Mean [SD], *n* = 3) of skeletal muscle EGFP^+^ cells. These findings vary to some extent from earlier reports indicating variation in Sca-1 expression that may be the consequence of *in vitro* expansion [2], [17].

Previous studies have established freshly-isolated muscle SP cells are negative for CD45, CD43, c-kit, CD11, Gr-1, B220, CD4 and CD8, while they remain positive for Sca-1, as do some MP cells [10]. Freshly-isolated bone marrow SP cells are typically positive for CD45, c-kit, and Sca-1 and negative for lineage markers. In addition, SP cells isolated from either source are negative for myogenic markers such as Pax7, Myf5, desmin, and MyoD [2], [18] as expression of these proteins is indicative of myogenic specification and differentiation. At present, Hoechst 33342 staining remains the standard means to distinguish the SP from non-SP cells. While the ABCG2 transporter is expressed by bone marrow and skeletal muscle SP cells, it is also present in many other cell types, thereby excluding it as a unique selection marker [19]. In addition, other ABC transporters can contribute to Hoechst efflux and expression of these proteins is likely regulated in a tissue- and developmental-specific manner [5]. Because our expansion cultures utilized myoblast feeder cells, we focused on studying the expression of established markers of muscle differentiation in both EGFP^high^ and EGFP^low^ fractions.

For immuno-staining experiments, we used antibodies directed against the ABCG2 transporter, the muscle-specific intermediate filament protein desmin, the satellite cell marker Pax7, the myogenic regulatory proteins MyoD and myogenin, as well as the Sca-1 antigen (Fig. 4C and 4D). EGFP^high^ fractions stained nearly exclusively for the ABCG2 transporter; whereas, EGFP^low^ cells stained similar to a mixed population of SP and MP cells. Specifically, skeletal muscle EGFP^low^ cells were positive for the muscle-specific marker desmin, whereas the same fraction from expanded bone marrow SP cells was, for the most part, not myogenic despite being cultured in the presence of myoblast feeders. Staining for the transiently-expressed transcription factors MyoD and myogenin indicated EGFP^low^ skeletal muscle cells were undergoing myogenic specification and differentiation. There were also a substantial number of cells from the EGFP^low^ fractions that stained for ABCG2, although they generally stained less intensely than EGFP^high^ cells. Obviously, some EGFP^low^ cells representing the SP fraction as well as some MP cells strongly-express ABCG2 [19], [20]. Together, our findings, in agreement with earlier studies, point to a gradual loss in ABCG2 as SP cells commit to a myogenic lineage [9]. These experiments provided evidence that the EGFP^high^ fraction was equivalent to the SP and increases throughout time in culture. This was true both within and between individual passages (Fig. 4E).

Since we have identified a method to reliably expand SP cells from bone marrow and skeletal muscle, an immediate goal will be to identify novel markers for isolating and purifying these cells without the need for fluorophores such as EGFP, Rhodamine 123, and Hoechst 33342. Ideally, these experiments will eliminate the need for flow cytometry to isolate expanded SP cells. This would also avoid exposing cells to potentially mutagenic substances, high-intensity UV light, and abolish a potential source of contamination. A second goal would be to replace C2C12 cells with MP cells as feeders. In the end, we anticipate that subsequent studies will determine the utility of fractionated SP cells as candidates for tissue regeneration and repair. The means to dependably expand the SP should foster additional experiments aimed to more fully ascertain the therapeutic potential of these cells.
